# Victimization and Perception of Abuse in Adolescent and Young Homosexual and Heterosexual Couples in Spain

**DOI:** 10.3390/healthcare11131873

**Published:** 2023-06-28

**Authors:** Noelia Aguilera-Jiménez, Luis Rodríguez-Franco, Francisco Javier Rodríguez-Díaz, Jose Ramón Alameda-Bailén, Susana G. Paíno-Quesada

**Affiliations:** 1 Department of Psychology, University of Oviedo, 33003 Oviedo, Spain; noelia.aguilera@dpee.uhu.es (N.A.-J.); gallego@uniovi.es (F.J.R.-D.); 2 Department of Personality, Assessment and Psychological Treatment, University of Seville, 41018 Seville, Spain; lurodri@us.es; 3 Department of Clinical and Experimental Psychology, University of Huelva, 21071 Huelva, Spain; alameda@uhu.es

**Keywords:** adolescent and young couple violence, homosexual couples, heterosexual couples, violent victimization, perception of abuse

## Abstract

Currently, violence in adolescent and young couples has a significant social impact on young people’s physical and psychological health. However, the study of violence in homosexual couples must also be addressed. This research analyzes the levels of violent victimization and the perception of abuse in both homosexual and heterosexual couples. Participants’ ages ranged between 14 and 29 years (*M* = 20.14, *SD* = 3.464). We used The Dating Violence Questionnaire-Revised (CUIVNO-R), which was applied in two consecutive studies. The results indicate high levels of victimization, especially in the sample of homosexual participants. The scores generally show a low perception of couple violence but high victimization rates. The results of this study reveal the importance of the issue of violence in couples from minority groups and suggest that couple violence should not be understood as unidirectional, i.e., exclusively from men to women. These findings show the need for education in healthy relationships and consideration of different types of couples in these relationships.

## 1. Introduction

### 1.1. The Importance of the Study of Violence in Adolescent Couples and Young Homosexuals and Heterosexuals in the Spanish Context

In the Spanish context, the term to refer to intimate partner violence at the legislative level does not currently include violence between homosexual men or homosexual women or within the LGTBIQ+ collective. Therefore, official published data on these minority groups are scarce, and more so when it comes to adolescent couples and young homosexuals. Given the lack of data and the scarcity of findings related to the prevalence of violence in the latter group [1,2,3], our study analyzes this problem that affects adolescents’ and young people’s health and social and psychological well-being [4]. The main justification is that, so far, the investigations have adopted a heteronormative viewpoint [5]; that is, they have focused on heterosexual couples and, above all, on the one-directional violence of men towards women. However, it must be understood that violence can be present in any relationship; it can place anyone at risk and, just as in heterosexual couples, men and women with a homosexual orientation frequently suffer abusive behaviors of the same typologies: physical, psychological, and sexual abuse in the relationship [6,7,8,9,10,11,12].

Spain does not have official statistical data that specifically indicate the percentages of complaints from this community sample and, much less, from young couples. It must be added that it is especially complex for this group to request resources for help and guidance [3,13]. The lack of any description of how people manage these situations makes it impossible to develop effective and beneficial programs and resources for these minority groups [14], hence, the importance of this issue. Thus, it is necessary to know their characteristics to propose adapted responses based on better-adjusted violence-prevention guidance, the identification of the different ways of performing or suffering violence at these life stages, and—considering the current novelties—teaching relationships based on respect and attending to the well-being of the self and the other person.

### 1.2. Reference Terminologies for Intimate Partner Violence Research

It is essential to understand the terminology used in heterosexual and homosexual couple violence and in the LGTBIQ+ collective. On the one hand, we start with the term most frequently referenced: intimate partner violence (IPV), which, according to the World Health Organization [15] (p. 1), is understood as behavior within an intimate relationship that includes acts of physical aggression (slapping, hitting, or kicking), psychological abuse (intimidation, belittling, or continuous humiliation), forced sexual interactions, and controlling behaviors (isolation from family and friends, control of the partner’s movements, and restriction of access to information or assistance). Another concept of interest is adolescent dating violence (DV), or Adolescent Dating Violence (ADV), or Teen Dating Violence (TDV). There are many definitions of these terms underlining the inclusion of intentional psychological, physical, and/or sexual abuse between people in a dating relationship [7]. DV has been studied for at least four decades [16]. In addition, the Centers for Disease Control and Prevention [17] adds that such abuse can be face-to-face or electronic, and Muñiz-Rivas et al. [18] indicate the importance of addressing online violence.

On the other hand, one of the terminologies to approach and better understand this neglected line of research regards violence performed in homosexual couples. In our context, the most consolidated term to refer to this is Intragender Violence (IV), which occurs in affective-sexual relationships between people of the same sex. It is the same as or similar to man-to-woman violence, with the intention of dominating and controlling the partner [19,20,21]. This stance generates discrepancy, as IV can refer to violence when one member of the couple is transgender, transsexual, intersexual, queer, or indeterminate, that is, couples within the LGTBIQ+ collective. In short, the term IV does not consider people or couples where one member belongs to the transsexual, transgender, or intersexual (TTI) collective and may be homosexual or heterosexual.

Understanding these terminologies requires identifying the differentiation offered in our legal context. Firstly, according to the Organic Law 1/2004 of December 28 on Comprehensive Protection Measures against Gender Violence [22], gender-based violence (or Intimate Partner Violence Against Women) is violence exercised only by the man to control or exercise power over the woman who is or has been a spouse or with whom he is or has been in a relationship with affective involvement, even without cohabitation. The exercise of power or the desire to control and dominate the other member of the affective relationship is shared in both GV (Gender-based Violence) and IV, but IV does not differentiate the sexes [19].

Finally, another confusing term is domestic violence (Law 27/2003, of July 31, regulating the order for the protection of victims of domestic violence) [23], which includes violence manifested in the family, previously or presently cohabitating, in which the victim can be either a man or a woman. However, although this excludes couples whose members are not spouses or cohabitants, it allows violence in homosexual couples to be legislated as domestic violence.

### 1.3. Contributions from the Literature on the Prevalence of Violence in Adolescent Couples and Young Homosexuals and Heterosexuals

Currently, IPV is a public health problem [8,9] affecting younger people in our society, as it begins at an early age [4,24]. Despite considering IV a global problem, this population has been neglected [25,26,27], highlighting the scarcity of published research, which is even more pronounced in our context.

The references on the prevalence of violence in homosexual couples reflect a high prevalence, with very significant percentages in the Spanish population [20,28]. This is also observed in the Latin American population, where both couple members are equally victims and perpetrators [29]. Other results are provided in the review by Edwards et al. [30], which indicated a higher prevalence among lesbian, gay, and bisexual people than among heterosexual people, consistent with other studies. Specifically, data on violence within homosexual couples show a prevalence range of 25.2–33.3% for men and 25–40.4% for women [31,32]. When specifying the typology, psychological violence reaches 63% for women and 60% for men [32]. Physical and sexual violence occurs among 44% of lesbians and 26% of gays [33]. Psychological victimization is the most frequent, followed by physical and sexual typologies [5,34]. In this regard, in Spain, Ortega [28], in his study on aggression in homosexual couples in a sample of young people aged 18 to 29, reports a prevalence of 13.09% for psychological violence, 4.57% for sexual violence, and 3.53% for physical violence, finding the highest rates of violent victimization in these minority groups for this age range.

Therefore, violence in this population seems to predispose it to a higher risk of mental health problems [25,35] and a higher probability of these individuals suffering violence in adulthood because they had suffered it in adolescence or youth [25,33]. There is also a lack of measures, evaluation instruments, and validations targeting these minorities, showing how urgent it is to create them [1].

### 1.4. Perception of Abuse in Adolescent Couples and Young Homosexuals and Heterosexuals

Finally, a little-studied but interesting phenomenon that has been neglected among these minorities is the perception of abuse by their partners, which has been studied in heterosexual couples [36,37,38,39]. This perception consists of a person’s thoughts and cognitions when feeling abused by another person. In the Spanish population, many authors state that it is necessary to study not only the perception of being mistreated by one’s partner but also the perception of stalking or the resulting feeling of fear, especially if the victim feels trapped in their relationship. This is closely connected to victimization through coercive behaviors or other more subtle forms of violence [37,38,40]. All this provides information about the characteristics of people who do not recognize or identify themselves as victims. Considering these dissonances, many researchers have highlighted the relevance of what is called “unperceived abuse-unperceived violence” or “technical abuse” [37,38,39,40,41,42]. It is interesting to note the results obtained by Gutiérrez Prieto et al. [43], who report that young people can recognize their partners’ inappropriate actions, facilitating their perception of violent abuse regarding these behaviors.

Therefore, IPV should be addressed in a way that integrates all the different perspectives [44]. This would represent an advancement at the social, educational, and legal levels, considering the behaviors that, although just as illegal, are penalized differently. It is therefore essential to provide data that highlight this problem to adapt prevention and intervention projects for homosexual couples and the LGTBIQ+ collective.

We propose, as a general objective, to verify the levels of violent victimization and the perception of abuse in adolescent and young couples, both homosexual and heterosexual, to determine the scope of the problem in these minority groups (hereafter, we will use the terms heterosexual, homosexual, lesbian for couples of women, and gays for couples of men). The specific objectives are:

1. To analyze the prevalence of and differences in violent victimization according to sexual orientation (heterosexuals and homosexuals (gays and lesbians)) and sex.
2. To describe the perception of abuse (*feeling afraid or trapped in the relationship or feeling mistreated*) according to sexual orientation.
3. To verify the prevalence of victimization according to the existence of the perception of abuse based on sexual orientation and to observe a possible correspondence between victimization and levels of perception of abuse.

## 2. Method

### 2.1. Participants

The sample consists of 552 students of Compulsory Secondary Education, High school, Vocational training, and university from different provinces of Spain. Of them, 46% (*n* = 254) were female, and 54% (*n* = 298) were male, and their ages ranged between 14 and 29 years (M = 20.14, *SD* = 3.464). Of the participants, 156 (28.3%) reported having had a same-sex partner and 396 (71.7%) reported having had a partner of a different sex. Regarding those who reported having had same-sex partners, 48.7% (*n* = 76) were female, and 51.3% (*n* = 80) were male. Of the participants with different-sex partners, 44.9% (*n* = 178) were female, and 55.1% (*n* = 218) male. By level of education, 61.2% (*n* = 338) were pre-university students (Compulsory Secondary Education, High school, and Vocational training studies), and 38.6% (*n* = 213) were university students. Of the total sample, 90.2% responded *Yes* to the question “*Do you have a partner at this time?*” and 9.8% answered negatively. Finally, 21% were working. Concerning religion, 32.4% were not at all religious, 47.9% were moderately religious, and 24.8% were very religious.

Two groups were created for this research. On the one hand, we selected the cases where the participants indicated they had had or had a different-sex partner. This selection was exported to a different database, and two random samples of exact cases were extracted (*n* = 198). On the other hand, we selected the cases of participants who reported having had a same-sex partner (*n* = 156). Subsequently, the cases were entered into two different databases, resulting in the two studies analyzing 354 people (Study 1 and Study 2 (S1 and S2, respectively)).

### 2.2. Procedure

This research was carried out following the Declaration of Helsinki. As a large part of the sample was minors, compliance with the ethical criteria of working with minor participants was ensured. First, we arranged a meeting at the schools of Compulsory Secondary Education to explain the objectives of the research. After acceptance by the school directors, we collected the data. Data protection was guaranteed at all times. We provided all the groups with information about the usefulness and the purpose of the research to determine and describe the interpersonal dynamics within couple relationships. The groups were invited to participate voluntarily and received no compensation for their participation. Before completing the instruments, we obtained all the participants’ informed consent and gave them the necessary instructions to complement the battery of instruments. This same procedure was carried out in the university centers.

This research used a non-probabilistic, intentional or judgment survey methodology because the sample was selected according to the requirements of the research goals.

### 2.3. Variables and Measurement Instruments

***Sociodemographic characteristics.*** Sociodemographic information was collected about the participant and their partner: age, sex, level of education, income of the family nucleus, employment status, and religious beliefs.

***Partner violence.*** We utilized the *Cuestionario de Violencia entre Novios*, *CUIVNO-R* [Dating Violence; Questionnaire] [2]. It comprises 20 items collecting information on victimization and perpetration of violence in young people’s dating relationships. These items present behaviors or situations of abuse that can occur within the relationship, and their frequency is rated on a 5-point Likert scale ranging from 0 (*never*) to 4 (*almost always*). The CUIVNO-R offers 5 different forms of DV: *detachment*, *humiliation*, *coercion*, *physical violence*, *and sexual violence*. Detachment violence is evaluated, among others, via the following item: “your couple does not speak to you or leaves for several days, without any explanation, in order to show annoyance”. Items regarding the other four DV forms include “violence through humiliation: your couple ridicules or insults you for the ideas you sustain”; “violence through coercion: your couple tests your love by setting up traps to see if you are cheating on your couple”; “sexual violence: you feel forced to comply certain sexual acts”; “physical violence: your couple has hit you”. Internal consistency for the five scales ranges from 0.89 to 0.97 (Cronbach alpha), and for the total scale *α* = 0.98. The internal consistency for the different scales of Study 1 ranged between 0.89 and 0.97 (Cronbach alpha) and for the total scale *α* = 0.98. In Study 2, the indices of the different scales ranged from 0.90 to 0.98, and for the total scale *α* = 0.98.

***Perception of abuse.*** Following the requirements of the United Nations in 2015 [45] and because of the indications after the Istanbul Convention in 2011 [46], modifications were introduced in the Macro-Survey on Violence against Women (2015) [47], especially those related to the appropriateness of more accurately identifying forms of violence. Therefore, additional information was collected about whether the participant had experienced stalking (continuous or repeated harassment, following, spying) leading to fear of their partner. This indicator has been shown to be extremely effective in the latest official macro-survey carried out in Spain in 2019 on violence against women [40] and has been verified in previous empirical studies [42]. Through a second question, the participant was asked about their feeling of being trapped in the relationship to determine their subjective perception of the possibility of their being able to leave their affective relationship. Finally, we examined the perception or awareness of the situation of abuse. Specifically, there were 3 dichotomous questions with a *Yes* or *No* response: “*Do you feel or have you ever felt afraid of your partner?*”, “*Do you feel or have you felt trapped in your relationship?*”, *and* “*Have you felt mistreated in your relationship?*”. The internal consistency for the total sample was α = 0.533 (Study 1, α = 0.544, and Study 2, α = 0.576).

## 3. Statistical Analysis

The IBM SPSS statistical package, version 27, was used for data analysis. Descriptive statistics of the relevant variables of the study were conducted, assuming in all the analyses the criterion of “tolerance 0”; that is, violent behavior is considered to be present when any of the items that measure violent victimization is responded to with a value different from 0.

**Prevalence of Violent Victimization in Adolescent Couples and Among Young Homosexuals and Heterosexuals.** First, the typologies of violence according to the sexual orientation of each study group (heterosexual–homosexual) were analyzed. For this purpose, the level of victimization in the couple was calculated using the five subscales of CUIVNO-R (detachment, humiliation, coercion, and sexual and physical violence). Subsequently, we performed a Student’s *t* contrast for independent samples and used the Bayes factor (BF) measures to determine the strength of the evidence of the differences. This same analysis was used considering the participants’ sex and sexual orientation. We also analyzed the samples of participants who reported having had heterosexual partners and the couples of women (lesbians) and men (gays) who indicated having had homosexual partners. For this purpose, a repeated-measures analysis of variance (*ANOVA*) was carried out between the analyzed groups. Subsequently, the Bonferroni adjustment was calculated separately for the analysis of each group.

**Perception of abuse in adolescent couples and among young homosexuals and heterosexuals.** Secondly, we proceeded to describe the perception of abuse presented by the participants in the analyzed sample (feeling fear in the relationship, feeling trapped, or feeling mistreated). To this end, a descriptive frequency analysis was carried out to determine the percentage of participants who had answered any of the three questions affirmatively. Total scores and scores according to the sexual orientation of the sample were obtained. Subsequently, we calculated the difference in proportions for this analysis [48,49].

**Violent Victimization and perception of abuse in adolescent couples and among young homosexuals and heterosexuals.** We created two groups after the analyses to verify the correspondence between the victimization observed and the participants’ perception of abuse. On the one hand, “Yes, Perception of Abuse” included the participants who had affirmatively answered any of the three questions on fear, entrapment, and abuse. The “No Perception of Abuse” comprised the participants who had answered all three questions negatively. In addition, the five factors of violence were transformed into dichotomous variables (0 = No Violence; 1 = Violence). Subsequently, “Total Violent Victimization” was obtained by adding the factors and transforming the variable to be dichotomous (0 = No Victimization; 1 = Victimization). After creating the groups, we determined the differences in their victimization rates of the five factors of violence of the CUIVNO-R and according to sexual orientation through chi-square (χ^2^). Accordingly, we identified the people who presented indicators of violence that did not perceive them as such, as in other empirical studies [38,42,50].

## 4. Results

Below, the results of the two research studies are described and presented conjointly for more clarity. The sample of participants who indicated having had homosexual partners was used in the two studies.

**Prevalence of violent victimization in adolescent couples and among young homosexuals and heterosexuals.** In order to observe the prevalence of victimization and determine the differences in violent victimization (typologies of violence) between the two samples analyzed (according to sexual orientation), Student’s t was performed to estimate the BF and calculate Cohen’s effect size. The results (Table 1) indicate that homosexual couples obtained higher and statistically significant scores in both Studies 1 and 2. Physical violence (hitting, pushing, shaking, throwing objects, etc.) [S1: *t*(346) = 38.35, *p* < 0.001, *d* = 2.88; S2: *t*(347) = 40.45, *p* < 0.001, *d* = 2.93], detachment (disappearing, ceasing to talk to, and ignoring the partner’s feelings) [S1: *t*(347) = 29.06, *p* < 0.001, *d* = 2.61; S2: *t*(349) = 34.78, *p* < 0.001, *d* = 2.81] and humiliation (insults and excessive criticism) [S1: *t*(345) = 32.24, *p* < 0.001, *d* = 2.46; S2: *t*(346) = 32.81, *p* < 0.001, *d* = 2.45] were the typologies with the greatest difference between the two samples, with effect sizes greater than 1, and the BF values reflect strong evidence of the obtained differences (*BF =* 0.000). To calculate the differences among heterosexual couples, gay couples, and lesbian couples, a one-factor *ANOVA* was performed. The corresponding repeated-measures *ANOVA* yielded significant group differences in the five types of violence identified in CUIVNO-R (Table 2). All typologies were significantly higher in the two groups of homosexual couples (gays and lesbians) than in the heterosexual group. However, when analyzing each group separately, the pairwise comparison with the Bonferroni adjustment showed significant differences between heterosexual couples and gay couples (*p* = 0.000) and between heterosexual couples and lesbian couples *(p* = 0.000) in both Study 1 and Study 2. On the other hand, we observed no significant differences between the groups of homosexual couples (gays and lesbians) except for sexual violence, which was significant in both studies [S1: *p* = 0.003; S2: *p* = 0.002].

Secondly, concerning the differences in violent victimization according to sex in both groups (homosexuals and heterosexuals), Table 3 shows statistically significant differences for all types of violence. Regarding the group of women, lesbians obtained the highest means, with the highest effect sizes in physical violence [S1: *t*(163) = 24.95, *p* < 0.001, *d* = 2.87; S2: *t*(163) = 27.19, *p* < 0.001, *d* = 2.91], violence due to detachment [S1: *t*(162) = 19.58, *p* < 0.001, *d* = 2.58; S2: *t*(163) = 22.28, *p* < 0.001, *d* = 2.70], and violence due to humiliation [S1: *t*(159) = 21.36, *p* < 0.001, *d* = 2.37; S2: *t*(160) = 21.16, *p* < 0.001, *d* = 2.35]. In men, the largest effect sizes were obtained for the typologies of physical violence [S1: *t*(181) *=* 29.49, *p* < 0.001, *d = 2.89*; S2: *t*(182) *=* 29.94, *p* < 0.001, *d =* 2.92] and detachment [S1: *t*(183) = 21.42, *p* < 0.001, *d* = 2.65; S2: *t*(184) = 26.99, *p* < 0.001, *d* = 2.91], followed by violence by humiliation [S1: *t*(184) = 24.21, *p* < 0.001, *d* = 2.56; S2: *t*(184) = 25.39, *p* < 0.001, *d* = 2.57] and coercion towards the partner [S1: *t*(185) = 20.02, *p* < 0.001, *d* = 2.33; S2: *t*(185) = 23.30, *p* < 0.001, *d* = 2.52], with gays again obtaining higher means. In addition, the Bayes factor (*BF* = 0.000) reflected strong evidence of the differences between homosexual and heterosexual women and men.

**Figure 1:**
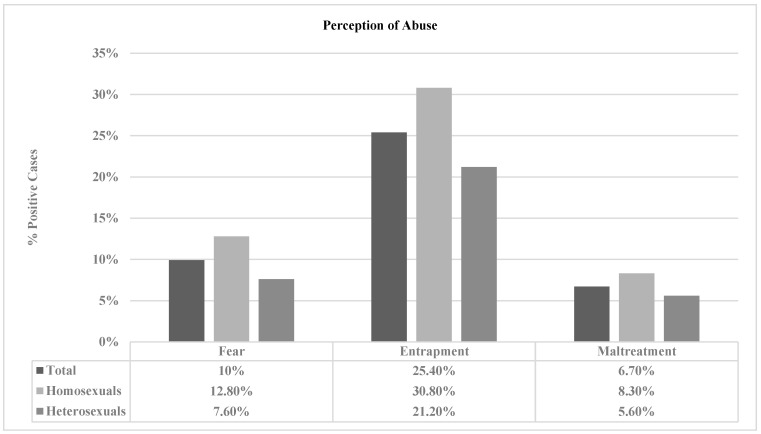
Percentages of positive cases of perception of abuse according to sexual orientation in the sample of Study 1.

**Figure 2:**
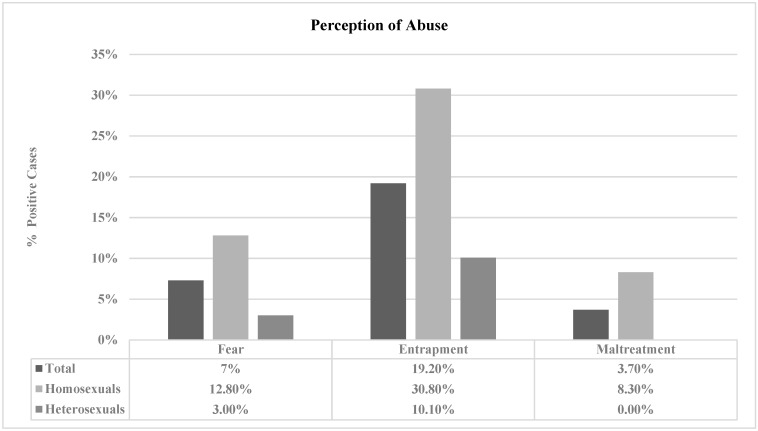
Percentages of positive cases of perception of abuse according to sexual orientation in the sample of Study 2.

**Perception of abuse in adolescent couples and among young homosexuals and heterosexuals.** Figure 1 and Figure 2 show that the feeling of being trapped in the relationship obtained the highest percentage for the total sample [S1: 25.40%; S2: 19.20%]. When analyzing the two groups, homosexual couples scored higher on all three questions in both studies. Moreover, significant scores were obtained when asking the participants about their feelings of entrapment in the relationship [S1: *p* = 0.025; S2: *p* = 0.001] and their fear of their partner [S2: *p* = 0.001] and feeling mistreated [S2: *p* = 0.001], although only in Study 2. Regarding the difference in proportions, the results indicated no significant differences between Study 1 and Study 2 in any of the responses concerning the perception of abuse (mistreatment, fear, and entrapment).

**Violent victimization and perception of abuse in adolescent couples and among young homosexuals and heterosexuals.** Table 4 provides the percentages of perceived abuse (“Yes Perception of Abuse”/“No Perception of Abuse”) in heterosexual and homosexual couples. Of the total sample, the percentage of participants who did not perceive abuse was higher in both studies [S1: *n* = 244 (68.90%); S2: *n* = 275 (77.70%)] than the percentage of those who perceived abuse [S1: *n* = 108 (30.50%); S2: *n* = 78 (22%)], with statistically significant differences [S1: *χ*^2^ = 3.87, *p* = 0.032; S2: *χ*^2^ = 31.61, *p* = 0.001]. We also observed higher violent victimization than non-victimization in the two studies. Specifically, we observed victimization scores ranging between 69.70% and 99.40% in contrast to non-victimization, which ranged between 0.60% and 30.30%. The presence of high levels of victimization and the high lack of perception of abuse are noteworthy. When inquiring about this, in both Studies 1 and 2, homosexual couples reached levels of victimization of 99.40%, whereas 63.50% of them perceived no abuse. Concerning the samples of heterosexual couples, in Study 1, 73.60% reported no perception of abuse, although 75.80% were victims of violence. In the case of Study 2, 88.90% did not perceive violence, although they presented victimization levels of 69.70%. Secondly, in the case of heterosexual couples, the chi-square revealed significant differences (in both studies) in all types of violence except for sexual and physical violence, which were nonsignificant in Study 2. The highest percentage of violence was observed in participants who had perceived abuse. However, this was not observed in the sample of homosexual couples, because only one case reported no victimization, whereas the remaining cases presented violent victimization with no differences among the typologies.

**Figure 3:**
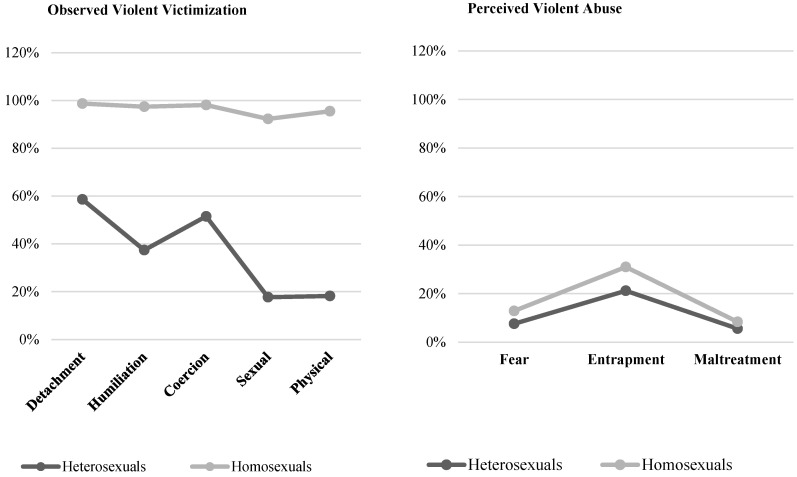
Visibility and invisibility of violent victimization and perception of abuse as a function of sexual orientation in Study 1.

**Figure 4:**
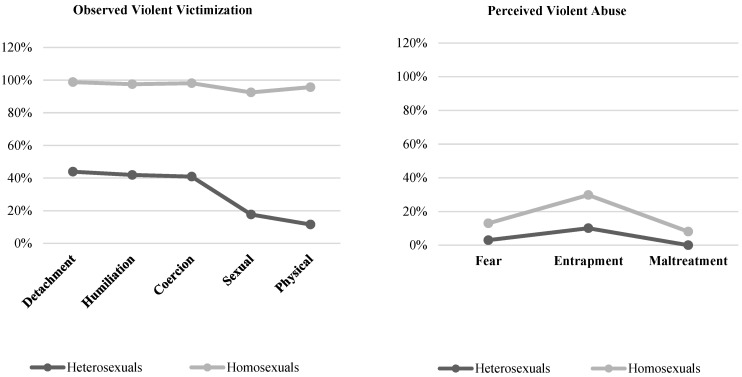
Visibility and invisibility of violent victimization and perception of abuse as a function of sexual orientation in Study 2.

Finally, we examined the possible correspondence between the violent victimization observed in the two studies and the levels of perception of abuse. As seen in Figure 3 and Figure 4, the high rates of violent victimization do not correspond to the perception of abuse indicated by the participants. Specifically, in homosexual couples (Studies 1 and 2), the percentage of victimization exceeded 90%, whereas the percentage of the perception of abuse did not exceed 31%. This mismatch also occurred among participants who reported having or having been heterosexual couples, although it was not as notable. In Study 1, violent victimization reached 59%, but the perception of violence only reached 21%. Consistent with the above, in Study 2, victimization also reached 44% compared to the 10% who had reported a perception of abuse. This highlights the invisibility of adolescents’ and young people’s suffering of violence.

## 5. Discussion

Violence is manifested in interpersonal relationships; specifically, this study shows that violence in homosexual couples is a problem of the same dimension as violence in heterosexual couples. Therefore, it is essential to ensure its adequate legal treatment considering all existing dimensions, like IPV. According to the approach and the findings, this study provides novel information on the extent of the problem of IPV in vulnerable groups. In light of our results, we underline the high frequency of IPV among both heterosexuals and homosexuals, showing the need to be aware that violence can appear in any interpersonal relationship. Consequently, we must educate youth in having care and respect for the other person and provide sexual education and sufficient information so that young people can identify the indicators of violence, which can affect their physical, mental, and social health.

First, based on the principle of “zero tolerance”, our results are consistent with other studies carried out in Spain and Latin America finding comparable rates of victimization among both homosexuals and heterosexuals—although higher among the former [20,28,29]—as well as with the data published in other countries, such as that of Edwards et al. [30]. Among homosexuals, physical victimization stands out, followed by other types of violence, such as psychological or unperceived violence; that is, detachment (disappearing or not talking or ignoring the partner’s feelings) and humiliation (insults or criticism of the partner). When comparing our findings on homosexual couples with other investigations, there are some discrepancies; in particular, a discrepancy is observed in the order in which the typologies of violence are presented. That is, physical violence, detachment, and humiliation of the partner are more frequent in our results than sexual violence and coercion, which are also present but with lower rates. In contrast, other studies, such as that of Ortega [28], report that in samples of adolescents and young people (from 14 to 29 years old), psychological violence obtains the highest percentage, followed by sexual and physical violence. The same is true of the results of Walters et al. [32], where psychological violence was more frequent than physical violence, or the results obtained by Martín-Storey [33], where physical and sexual violence predominated among these minorities. This is not observed in heterosexuals, whose members are mostly victims of violence due to detachment, coercion, and humiliation, in coherence with the findings of other studies where psychological violence prevails [10,37,38,39,42].

Despite this controversy, the analyses show the different types of violent victimization within couple relationships, highlighting the results among homosexuals [5,31,32,34]. In young heterosexual relationships, we confirm the high prevalence of psychological violence performed and suffered both by males and females, with detachment, coercion, and humiliation being common [10,38].

Another relevant finding of this study is the differences in all the typologies of violence (both in heterosexual and homosexual couples) as a function of the participants’ sex. Females suffer more physical victimization, detachment, and humiliation, mostly in homosexual couples. On the other hand, males repeat the typologies of physical violence, detachment, and humiliation, also adding coercion. Therefore, this shows that victimization can affect anyone. Hence, limiting the roles of female victims and male aggressors implies ignoring the existing reality because victimization affects a large part of the population [51]. Thus, the predominance of humiliating one’s partner, especially in homosexual couples, reveals what the LGTBIQ+ collective refers to when they talk about the threat of “coming out of the closet” or “outness”. This threat is aimed at controlling or retaining the other member of the relationship by threatening them with society’s ridicule and contempt. This phenomenon suffered by these minorities promotes tension and stress in the relationship dynamic, turning it negative [52]. The findings of violence exercised through coercion, which is more predominant in males, are consistent with the meta-analysis of Spencer et al. [53], which reports that this typology of violence is a risk factor among young men. On the other hand, according to the data on physical violence, victimization in homosexual relationships, regardless of sex, is striking, following research indicating that males are perpetrators of physical violence and females of mild physical violence [38,54].

Summing up, we can state that our adolescent and young populations are immersed in violent relationships, with a tendency to perpetuate and normalize the dynamics of conflictive relationships [55], and two issues are essential: (1) violence at this stage can become violence in adulthood [24,25,33]; and (2) violence puts the physical and mental health of our younger population at risk [4,8,9].

Thirdly, with the aim of complying with the recommendations of the European Council [46] and the Macro-Survey on Violence against Women of 2015 and 2019 [40,47], we investigated the different ways of experiencing violence. We found percentages ranging from 3.70% to 25.40%, with homosexual couples reporting feeling these indicators to a greater extent. In particular, we emphasize that the feeling of being trapped in a relationship is the most frequent in the adolescent and young populations. This information is relevant for the study and future prevention as it can indicate emotional dependence on one’s partner, which correlates with negative coping in conflictive situations [41]. On the other hand, we found that fear of one’s partner presents percentages around 7–10%, indicating the possible existence of concealed cases of partner violence. These data are similar to those published by the 2019 Macro-Survey [40], suggesting that 13.90% of females aged 16 and older feared their partners. The perception of abuse in the relationship is shown to have the lowest prevalence. This is probably due to the difficulty of recognizing oneself as someone who is in an abusive or violent situation [39]. Similarly, this may also be due to the reductionist interpretation of psychological violence or unperceived violence, which poses a clear risk because it is considered a predictor of the partner’s physical violence [38,39,40,41,42,56].

On the other hand, regarding the third objective, we verified an evident lack of perception of abuse and high levels of victimization. These data generate alarm and should be addressed in prevention programs, and we highlight that of the total sample of homosexual couples, only one person was not victimized. Finally, from a global point of view, the couples in this research had difficulty in perceiving abuse because the percentage of “Perception of Abuse” was small (S1: 30.5%; S2: 22%) in contrast to the high levels of victimization observed. The non-perception of violence in heterosexual couples has already been detected in different studies [38,39,40,41,42,43]. A higher percentage of homosexual couples than heterosexual couples fails to recognize suffering violence as such; like other research, the data indicate a limited level of awareness of homosexual violence [36].

All the above leads us to consider that anyone can suffer violence within a couple relationship [6]. Although there are indeed differences between homosexual and heterosexual couples, this only indicates that victimization also exists within these couples, and there may even be a lack of perception of abusive acts. Thus, violence is present in our affective relationships and continues to be a worldwide problem affecting the public health of our adolescents and young people globally [8,9,11,12]. These results serve to propose changing the unique view that risk is only present in heterosexual couples because there is risk among men and women who have same-sex partners and in relationships where one member is transsexual, intersexual, queer, or indeterminate; in short, this risk exists for people who have interpersonal relationships with other people.

## 6. Limitations

Regarding the main limitations of this research, we point out the sample size. It would be interesting to increase it to generalize the results of violent victimization for our context. Samples of people with different sexual orientations or gender identities should be obtained to determine the differences due to maturational development according to age. The second limitation refers to the absence of data on the perpetration of violence. Integrating this variable would enable us to know if the victim is also an aggressor; that is, we could analyze the bi-directionality of violence, mainly in homosexual couples.

## 7. Conclusions

In conclusion, there is a need for changes in public and social policies so that the victims of violence have social resources. Regardless of whether they are heterosexual or of the LGTBIQ+ collective, this issue is still a social emergency. Neither the LGTBIQ+ nor the heterosexual populations seek IPV-related support services. In the former case, this is because of the barriers to receiving care [57], and, in the latter case, because of the difficulty of perceiving their relationship as abusive and, therefore, themselves as victims [38].

From this point of view, violence in homosexual couples must be considered a problem with the same dimensions as violence in heterosexual couples, and, therefore, resources must be adapted to the needs of this minority group [26]. In other words, it is necessary to apply social and egalitarian measures, educational measures that promote respect, and legal measures in which the system endorses and performs comprehensive evaluations where both members of the couple (victims and aggressors) are assessed without establishing differences. In short, defending the existence of other types of violence does not imply making gender differences invisible but rather avoids limiting violence exclusively to gender differences [44].

Finally, regarding future lines of research, we confirm the importance of addressing this area of prevention, given the results of different investigations on unperceived violence and high levels of violent victimization, with the justification of violence as a risk factor for victimization [58]. In this way, we can identify the indicators present in conflictive situations where inadequate coping strategies are implemented, generating abusive behaviors that are fully normalized. Therefore, we should continue to perform informative prevention, talk to young people, and conceptualize all the possible present and future situations they may encounter so that they can respond adequately for their well-being and that of others. Another critical point recommended by the United Nations [45] is the need to ask women if they have ever felt afraid of their current partner or their ex-partners. Our contributions indicate that not only should one ask women about fear, but exploring the feeling of entrapment and mistreatment in anyone within an interpersonal relationship is also relevant. Secondly, psychological proposals should be offered to create judicial measures of violence in homosexual couples to increase their sense of security and society’s protection towards these minority groups. Thirdly, there is a need for a working approach comparable to that used with heterosexual couples that analyzes not only the level of victimization but also the level of perpetration of violence, that is, that addresses bi-directional violence in same-sex couples, as noted above in the limitations. Finally, we should not ignore the need to develop specific and effective action plans because bi-directional violence may be a common pattern in relationships [38].

## Figures and Tables

**Table 1:** Violent Victimization in adolescent and young couples based on sexual orientation in Study 1 and Study 2.

Sexual Orientation/ Typologies of Violent Victimization		Heterosexuals		Homosexuals					
		*n*	$\bar{x}$	*n*	$\bar{x}$	*BF*	*t*	*df*	*d*
Detachment	S1	196	1.86	153	11.75	0.000	29.06 ***	347	2.61
	S2	198	1.12	153	11.75	0.000	34.78 ***	349	2.81
Humiliation	S1	197	0.70	150	11.29	0.000	32.24 ***	345	2.46
	S2	198	0.73	150	11.29	0.000	32.81 ***	346	2.45
Coercion	S1	196	1.55	153	10.18	0.000	26.26 ***	347	2.23
	S2	198	0.85	153	10.18	0.000	31.38 ***	349	2.41
Sexual	S1	197	0.38	149	10.56	0.000	27.57 ***	344	2.04
	S2	196	0.24	149	10.56	0.000	28.60 ***	343	2.07
Physical	S1	197	0.36	151	12.85	0.000	38.35 ***	346	2.88
	S2	198	0.21	151	12.85	0.000	40.45 ***	347	2.93

Note. *n*: number of subjects; $\bar{x}$: mean; *BF*: Bayes factor; *t*: Student’s t; *df*: degrees of freedom; *d*: Cohen’s *d*; S1: results Study 1; S2: results Study 2; *** *p* < 0.001.

**Table 2:** Repeated-measures analysis of variance of violent victimization of the groups in Study 1 and Study 2.

Sexual Orientation/ Typologies of Violent Victimization		Heterosexuals	Homosexuals		ANOVA
			Gay	Lesbians	
		$\bar{x}$	$\bar{x}$	$\bar{x}$	*F*
Detachment	S1	1.86	11.57	11.93	421.89 ***
	S2	1.12	11.57	11.93	604.43 ***
Humiliation	S1	0.70	11.08	11.52	520.00 ***
	S2	0.73	11.08	11.52	538.33 ***
Coercion	S1	1.55	9.94	10.43	345.293 ***
	S2	0.85	9.94	10.43	493.20 ***
Sexual	S1	0.38	9.63	11.47	396.887 ***
	S2	0.24	9.64	11.47	427.33 ***
Physical	S1	0.36	12.55	13.14	737.357 ***
	S2	0.21	12.55	13.14	820.39 ***

Note. $\bar{x}$: mean; *ANOVA*: repeated-measures ANOVA; *F*: F Test; S1: results Study 1; S2: results Study 2; *** *p* < 0.001.

**Table 3:** Violent Victimization in adolescent and young couples based on sex and sexual orientation in Study 1 and Study 2.

Typologies of Violent Victimization/Sex/Sexual Orientation		*n*	$\bar{x}$ FHeterosexual	*n*	$\bar{x}$ FHomosexual	*BF*	*t*	*df*	*d*	*n*	$\bar{x}$ MHeterosexual	*n*	$\bar{x}$ MHomosexual	*BF*	*t*	*df*	*d*
Detachment	S1	88	1.74	76	11.93	0.000	19.58 ***	162	2.58	108	1.95	77	11.57	0.000	21.42 ***	183	2.65
	S2	89	1.24	76	11.93	0.000	22.28 ***	163	2.70	109	1.02	77	11.57	0.000	26.99 ***	184	2.91
Humiliation	S1	88	0.59	73	11.52	0.000	21.36 ***	159	2.37	109	0.79	77	11.08	0.000	24.21 ***	184	2.56
	S2	89	0.67	73	11.52	0.000	21.16 ***	160	2.35	109	0.78	77	11.08	0.000	25.39 ***	184	2.57
Coercion	S1	87	1.36	75	10.43	0.000	17.26 ***	160	2.16	109	1.70	78	9.93	0.000	20.02 ***	185	2.33
	S2	89	0.65	75	10.43	0.000	21.13 ***	162	2.33	109	1.02	78	9.94	0.000	23.30 ***	185	2.52
Sexual	S1	89	0.45	75	11.47	0.000	19.54 ***	162	2.16	108	0.32	74	9.63	0.000	19.77 ***	180	1.96
	S2	88	0.30	75	11.47	0.000	20.33 ***	161	2.19	108	0.20	74	9.64	0.000	20.44 ***	180	1.99
Physical	S1	89	0.36	76	13.14	0.000	24.95 ***	163	2.87	108	0.36	75	12.55	0.000	29.49 ***	181	2.89
	S2	89	0.17	76	13.14	0.000	27.19 ***	163	2.91	109	0.25	75	12.55	0.000	29.94 ***	182	2.92

Note. *n*: number of subjects; $\bar{x}$: mean; *BF*: Bayes factor; *t*: Student’s t; *df*: degrees of freedom; *d*: Cohen’s *d*; *F*: female; *M*: male; S1: results Study 1; S2: results Study 2; *** *p* < 0.001.

**Table 4:** Levels of violent victimization and perception of abuse among homosexuals and heterosexuals in Study 1 and Study 2.

			Heterosexuals			Homosexuals		
			“Yes Perception of Abuse”	“No Perception of Abuse”		“Yes Perception of Abuse”	“No Perception of Abuse”	
There is Victimization			***n*** **(%)**	***n*** **(%)**	***χ*** **^2^**	***n*** **(%)**	***n*** **(%)**	***χ*** **^2^**
	Detachment	S1	41(78.80%)	74(51.40%)	12.18 ***	59(100%)	97(98%)	1.15
		S2	16(72.70%)	71(40.30%)	8.33 **	59(100%)	97(98%)	1.15
	Humiliation	S1	26(50%)	48(33.10%)	4.66 *	56(100%)	95(96%)	2.32
		S2	17(77.30%)	66(37.50%)	12.71 ***	56(100%)	95(96%)	2.32
	Coercion	S1	41(78.80%)	60(41.40%)	21.50 ***	56(100%)	96(97%)	1.73
		S2	14(63.60%)	67(38.10%)	5.29 *	56(100%)	96(97%)	1.73
	Sexual	S1	18(34.60%)	17(11.70%)	13.73 ***	54(96.4%)	89(89.90%)	2.13
		S2	7(31.80%)	28(15.90%)	3.40	54(96.4%)	89(89.90%)	2.13
	Physical	S1	19(36.50%)	17(11.70%)	15.78 ***	55(98.2%)	93(93.90%)	1.52
		S2	5(22.70%)	18(10.20%)	2.98	55(98.2%)	93(93.90%)	1.52
Total Perception of Abuse		S1	52(26.30%)	145(73.20%)		56(35.9%)	99(63.50%)	
		S2	22(11.10%)	176(88.90%)		56(35.9%)	99(63.50%)	
Total Violent Victimization			**There is Victimization**	**No Victimization**		**There is Victimization**	**No Victimization**	
			***n*** **(%)**	***n*** **(%)**		***n*** **(%)**	***n*** **(%)**	
		S1	150(75.80%)	48(24.20%)	16.14 ***	155(99.4%)	1(0.60%)	0.60
		S2	138(69.70%)	60(30.30%)	3.255	155(99.4%)	1(0.60%)	0.60

Note. *N*: number of subjects; %: percentage of subjects; *χ*^2^: chi-square; S1: results Study 1; S2: results Study 2; *F*: female; *M*: male; * *p* < 0.05; ** *p* < 0.01; *** *p* < 0.001.

## Data Availability

The data reported here are available at request by scientific community members.

## References

[1] Barrientos J., Rodríguez-Carballeira A., Escatín J., Longares L. (2016). Violencia en parejas del mismo sexo: Revisión y perspectivas actuals [Violence in same-sex couples: Review and current perspectives]. Rev. Argent. Clín. Psicol..

[2] Rodríguez-Díaz F.J., Herrero J., Rodríguez-Franco L., Bringas-Molleda C., Paíno-Quesada S.G., Pérez B. (2017). Validation of Dating Violence Questionnaire-R (DVQ-R). Int. J. Clin. Health Psychol..

[3] Rojas-Solís J.L., Rojas I., Meza R.N., Villalobos A. (2021). Violencia de parejas gays y en hombres que tienen sexo con hombres: Una revisión sistemática exploratoria [Violence by gay partners and men who have sex with men: An exploratory systematic review]. Rev. Crim..

[4] Barroso-Corroto E., Cobo-Cuenca A.I., Laredo-Aguilera J.A., Santacruz-Salas E., Pozuelo-Carrascosa D.P., Rodríguez-Cañamero S., Martín-Espinos N.M., Carmona-Torres J. (2022). Dating violence, violence in social networks, anxiety and depression in nursing degree students: A cross-sectional study. J. Adv. Nurs..

[5] Gómez F., Barrientos J., Guzmán M., Cárdenas M., Bahamondes J. (2017). Violencia de pareja en hombres gay y mujeres lesbianas chilenas: Un estudio exploratorio [Intimate partner violence in Chilean gay men and lesbian women: An exploratory study]. Interdisciplinaria.

[6] Loinaz I., Ortiz-Tallo M., Sánchez L.M., Ferragut M. (2011). Clasificación multiaxial de agresores de pareja en centros penitenciarios [Multiaxial classification of partner aggressors in prisons]. Int. J. Clin. Health Psychol..

[7] Taquette S.R., Monteiro D.L.M. (2019). Causes and consequences of adolescent dating violence: A systematic review. J. Inj. Violence Res..

[8] García-Díaz V., Lana-Pérez A., Fernández-Feito A., Bringas-Molleda C., Rodríguez-Franco L., Rodríguez-Díaz F.J. (2018). Actitudes sexistas y reconocimiento del maltrato en parejas jóvenes [Sexist attitudes and recognition of abuse in young couples]. Aten. Prim..

[9] Heyman R.E., Kogan C.S., Foran H.M., Burns S.C., Slep A.M.S., Wojda A.K., Keeley J.W., Rebello T.J., Reed G.M. (2018). A case-controlled field study evaluating ICD-11 proposals for relational problems and intimate partner violence. Int. J. Clin. Health Psychol..

[10] Aguilera-Jiménez N., Rodríguez-Franco L., Rohlfs Domínguez P., Alameda Bailén J.R., Paíno-Quesada S.G. (2021). Relationships of adolescent and young couples with violent behaviors: Conflict resolution strategies. Int. J. Environ. Res. Public Health.

[11] Ferrer-Pérez V.A., Bosch-Fiol E. (2019). El Género en el Análisis de la Violencia Contra las Mujeres en la Pareja: De la “Ceguera” de Género a la Investigación Específica del Mismo [Gender in the Analysis of Intimate Partner Violence against Women: From Gender “Blindness” to Gender-Specific Research]. Anuario de Psicología Jurídica.

[12] Rubio-Garay F., López-González M.A., Carrasco M.A., Amor P.J. (2017). Prevalencia de la violencia en el noviazgo: Una revisión sistemática [Prevalence of dating violence: A systematic review]. Pap. Psicol..

[13] Rojas-Solís J.L., Guzmán-Pimentel M., Jiménez-Castro P., Martínez-Ruiz L., Flores-Hernández B.G. (2019). La violencia hacia los hombres en la pareja heterosexual: Una revisión de revisions [Violence against men in heterosexual couples: A review of reviews]. CyS.

[14] Kubicek K., McNeeley M., Collins S. (2015). “Same-sex relationship in a straight world”: Individual and societal influences on power and control in young men’s relationships. J. Interpers. Violence.

[15] World Health Organization (2013). Comprender y Abordar la Violencia Contra las Mujeres. Violencia Infligida por la Pareja 2013 [Understanding and Addressing Violence against Women. Intimate Partner Violence 2013].

[16] Exner-Cortnes D., Wolfe D.A., Temple J.R. (2018). Chapter13-Measuring adolescent dating violence. Adolescent Dating Violence: Theory, Research, and Prevention.

[17] Centers for Disease Control and Prevention (2021). Preventing Teen Dating Volence. What Is Teen Dating Violence?. https://www.cdc.gov/violenceprevention/intimatepartnerviolence/teendatingviolence/fastfact.html.

[18] Muñiz-Rivas M., Suárez-Relinque C., Estévez E., Povedano-Díaz A. (2023). Víctimas de violencia de pareja en la adolescencia: El papel del uso problemático de las redes sociales virtuales, la soledad y el clima familia [Victims of intimate partner violence in adolescence: The role of problematic use of virtual social networks, loneliness and family climate]. Anu. Psicol..

[19] Aldarte, Centro de Atención a Gays, Lesbianas y Transexuales (2012). Por los Buenos Tratos en las Relaciones Lésbicas y Homosexuales. Informe para la Inclusión de la Perspectiva LGTB en los Planteamientos Sobre Violencia de Género: Propuestas para el Debate [“For Good Treatment in Lesbian and Homosexual Relationships”. https://www.aldarte.org/comun/imagenes/documentos/BUENOSTRATOS.pdf.

[20] Aldarte, Centro de Atención a Gays, Lesbianas y Transexuales (2010). Estudio Sobre Violencia Intragénero. Informe Encuesta Violencia intragénero [“Study on Intragender Violence. Intragender Violence Survey Report”]. http://www.aldarte.org/comun/imagenes/documentos/informeencuestaviolenciaintragenero.pdf.

[21] Rodríguez Otero L.M., Lameiras M., Carrera M.V. (2017). Violencia en parejas gays, lesbianas y bisexuales: Una revisión sistemática 2002–2012 [Violence in gay, lesbian and bisexual couples: A systematic review]. Comunitaria.

[22] Ley Orgánica 1/2004, de 28 de diciembre, de Medidas de Protección Integral contra la Violencia de Género. https://www.boe.es/eli/es/lo/2004/12/28/1/con.

[23] Ley 27/2003, of July 31, Reguladora de la Orden de Protección de las Víctimas de la Violencia Doméstica. https://www.boe.es/eli/es/l/2003/07/31/27.

[24] Carrascosa L., Cava M.J., Buelga S. (2018). Perfil psicosocial de adolescentes españoles agresores y víctimas de violencia de pareja [Psychosocial profile of Spanish adolescent aggressors and victims of intimate partner violence]. Univ. Psychol..

[25] Gillum T.L. (2017). Adolescent dating violence experiences among sexual minority youth and implications for subsequent relationship quality. Child. Adolesc. Soc. Work. J..

[26] Laskey P., Bates E.A., Taylor J.C. (2019). A systematic literature review of intimate partner violence victimization: An inclusive review across gender and sexuality. Aggress. Violent. Behav..

[27] Trombetta T., Rollè L. (2022). Intimate partner violence perpetration among sexual minority people and associated factors: A systematic review of quantitative studies. Sex. Res. Social. Policy.

[28] Ortega A. (2014). Agresión en parejas homosexuales en España y Argentina: Prevalencias y heterosexismo [Aggression in homosexual couples in Spain and Argentina: Prevalences and heterosexism]. Ph.D. Thesis.

[29] Romero-Méndez C.A., Gómez M.J., Romo-Tobón R.J., Rojas-Solís J.C. (2020). Violencia en la pareja en jóvenes mexicanos del mismo sexo: Un estudio exploratorio [Intimate partner violence in Mexican same-sex youth: An exploratory study]. ACADEMO.

[30] Edwards K.M., Sylaska K.M., Neal A.M. (2015). Intimate partner violence among sexual minority population: A critical review of the literature agenda for future research. Psychol. Violence.

[31] Taylor N.T.B., Herman J.L. (2015). Intimate Partner Violence and Sexual Abuse among LGBT People: A Review of Existing Research. https://williamsinstitute.law.ucla.edu/wp-content/uploads/IPV-Sexual-Abuse-Among-LGBT-Nov-2015.pdf.

[32] Walters M.L., Chen J., Breiding M.J. (2013). The National Intimate Partner and Sexual Violence Survey (NISVS): 2010 Findings on Victimization by Sexual Orientation.

[33] Martin-Storey A. (2015). Prevalence of dating violence among sexual minority youth: Variation across gender, sexual minority identity and gender of sexual partners. J. Youth Adolesc..

[34] Barrientos J., Escartín J., Longares L., Rodríguez-Caballeira A. (2018). Sociodemographic characteristics of gay and lesbian victims of intimate partner psychological abuse in Spain and Latin America. Int. J. Soc. Psychol..

[35] Rodríguez J.G., Momeñe J., Olave L., Estévez A., Iruarrizaga I. (2019). La Dependencia Emocional y la Resolución de Conflictos en Heterosexuales, Homosexuales y Bisexuals [Emotional Dependence and Conflict Resolution in Heterosexuals, Homosexuals, and Bisexuals]. RED.

[36] Bornstein D.R., Fawcett J., Sullivan M., Senturia K.D., Shiu-Thornton S. (2006). Understanding the experiences of lesbian, bisexual and trans survivors of domestic violence: A qualitative study. J. Homosex..

[37] López-Cepero J., Lana A., Rodríguez-Franco L., Paíno S.G., Rodríguez-Díaz F.J. (2015). Percepción y etiquetado de la experiencia violenta en las relaciones de noviazgo juvenile [Perception and labeling of violent experience in youth dating relationships]. Gac. Sanit..

[38] Paíno-Quesada S.G., Aguilera-Jiménez N., Rodríguez-Franco L., Rodríguez-Díaz F.J., Alameda-Bailén J.R. (2020). Adolescent conflict and young adult couple relationships: Directionality of violence. Int. J. Psychol. Res..

[39] López-Cepero J., Rodríguez-Franco L., Rodríguez-Díaz F.J., Bringas-Molleda C., Paíno S.G. (2015). Percepción de la victimización en el noviazgo de adolescentes y jóvenes españoles [Perception of victimization in the dating relationships of Spanish adolescents and young people]. Rev. Iberoam. Psicol. Salud..

[40] Delegación del Gobierno para la Violencia de Género (2020). Subdirección General de Sensibilización, Prevención y Estudios de la Violencia de Género. Macroencuesta de Violencia contra la Mujer 2019 [Macro-Survey of Violence against Women 2019]. https://violenciagenero.igualdad.gob.es/violenciaEnCifras/macroencuesta2015/pdf/Macroencuesta_2019_estudio_investigacion.pdf.

[41] Martín B., Moral M.V. (2019). Relación entre dependencia emocional y maltrato psicológico en forma de victimización y agresión en jóvenes [Relationship between emotional dependence and psychological abuse in the form of victimization and aggression in young people]. Rev. Iberoam. Psicol. Salud..

[42] Rodríguez-Franco L., López-Cepero L., Rodríguez-Díaz F.J., Bringas Molleda C., Estrada Pineda C., Antuña Bellerín M.A., Quevedo-Blasco R. (2012). Labeling dating abuse. Undetected abuse among Spanish adolescents and young adults. Int. J. Clin. Health Psychol.

[43] Gutiérrez Prieto B., Bringas-Molleda C., Tornavacas Amado R. (2022). Autopercepción de maltrato y actitudes ante la victimización en las relaciones interpersonales de pareja [Self-perception of abuse and attitudes towards victimization in interpersonal relationships]. Anu. Psicol..

[44] Echeburúa E. (2019). Sobre el papel del género en la violencia de pareja contra la mujer. Comentario a Ferrer-Pérez y Bosch-Fiol, 2019 [On the role of gender in intimate partner violence against women. Comment to Ferrer-Pérez y Bosch-Fiol, 2019]. Anu. Psicol. Jurídica.

[45] United Nations (2015). Directrices para la producción de estadísticas sobre la violencia contra la mujer: Encuesta estadística. [Guidelines for the Production of Statistics on Violence against Women. Statistical Surveys]. https://oig.cepal.org/sites/default/files/directrices_estadisticas_violencia_contra_la_mujer.pdf.

[46] Comisión Europea Convenio del Consejo de Europa sobre Prevención y Lucha contra la Violencia contra las Mujeres y la Violencia Doméstica. Estambul, Turquía [Council of Europe Convention on the Prevention and Fight against Violence against Women and Domestic Violence]. https://rm.coe.int/1680462543.

[47] Delegación Gobierno para la Violencia de Género (2015). Macroencuesta de Violencia contra la Mujer 2015 [Macro-Survey of Violence against Women 2015]. https://violenciagenero.igualdad.gob.es/violenciaEnCifras/estudios/colecciones/pdf/Libro_22_Macroencuesta2015.pdf.

[48] Moore D.S., McCabe G.P., Freeman W.H. (2003). Introduction to the Practice of Statistics.

[49] Pryce G. (2005). Inference and Statistics in SPSS: A Course for Business and Social Science.

[50] Rodríguez-Franco L., Gracia C., Juarros-Baterretxea J., Fernández-Suárez A., Rodríguez-Díaz F.J. (2017). Agresores generalistas y especialistas en violencia de parejas jóvenes y adolescentes: Implicaciones en la implementación de los programas de prevención [Generalist and specialist batterers in teen and young dating violence: Implications for the development of prevention programs]. Acción Psicol..

[51] Rojas-Solís J.L., Romero-Méndez C.A. (2022). Violencia en el noviazgo: Análisis sobre su direccionalidad, percepción, aceptación, consideración de gravedad y búsqueda de apoyo [Dating violence: Analysis of its directionality, perception, acceptance, consideration of severity and search for support]. Health Addict..

[52] Feinstein B.A., McConnell E., Dyar C., Mustanski B., Newcomb M.E. (2019). Minority stress and relationship functioning among young male same-sex couples: An examination of actor-partner interdependence models. J. Consult. Clin. Psychol..

[53] Spencer C., Toews M., Anders K., Emanuels S. (2019). Risck Markers for physical teen dating violence perpetration: A meta-analysis. Trauma Violence Abuse.

[54] Gracia-Leiva M., Puente-Martínez A., Ubillos-Landa S., Páez-Rovira D. (2019). La violencia en el noviazgo (VN): Una revisión de meta-análisis [Dating violence (DV): A meta-analysis review]. An. Psicol..

[55] Bonache H., Ramírez-Santana G., González-Méndez R. (2016). Conflict resolution styles and teen dating violence. Int. J. Clin. Health Psychol..

[56] Juarros-Baterretxea J., Overall N., Herrero J., Rodríguez-Díaz F.J. (2019). Considering the effect of sexism on psychological intimate partner violence: A study with imprisoned men. Eur. J. Psych. Appl. Legal Context..

[57] Scheer J.R., Martín-Storey A., Baams L., Russell B. (2020). Help-seeking barriers among sexual and gender minority individuals who experience intimate partner violence victimization. Intimate Partner Violence and the LGBT+ Community.

[58] Galdo-Castiñeiras J.A., Hernández-Morante J.J., Morales-Moreno I., Echevarría-Pérez P. (2023). Educational intervention to decrease justification of adolescent dating violence: A comparative quasi-experimental study. Healthcare.

